# High Sensitivity Refractive Index Sensor Based on the Excitation of Long-Range Surface Plasmon Polaritons in H-Shaped Optical Fiber

**DOI:** 10.3390/s20072111

**Published:** 2020-04-09

**Authors:** Nelson Gomez-Cardona, Erick Reyes-Vera, Pedro Torres

**Affiliations:** 1 Escuela de Física, Universidad Nacional de Colombia, sede Medellín, Medellín 050034, Colombia; pitorres@unal.edu.co; 2 Departmento de Electrónica y Telecomunicaciones, Instituto Tecnológico Metropolitano, Medellín 050013, Colombia; erickreyes@itm.edu.co

**Keywords:** refractive index (RI) sensor, optical fiber sensor, optical waveguides, surface plasmon polariton (SPP), surface plasmon resonance

## Abstract

In this paper, we propose and numerically analyze a novel design for a high sensitivity refractive index (RI) sensor based on long-range surface plasmon resonance in H-shaped microstructured optical fiber with symmetrical dielectric–metal–dielectric waveguide (DMDW). The influences of geometrical and optical characteristics of the DMDW on the sensor performance are investigated theoretically. A large RI analyte range from 1.33 to 1.39 is evaluated to study the sensing characteristics of the proposed structure. The obtained results show that the DMDW improves the coupling between the fiber core mode and the plasmonic mode. The best configuration shows 27 nm of full width at half maximum with a resolution close to 1.3 × 10${}^{-5}$ nm, a high sensitivity of 7540 nm/RIU and a figure of merit of 280 RIU${}^{-1}$. Additionally, the proposed device has potential for multi-analyte sensing and self-reference when dissimilar DMDWs are deposited on the inner walls of the side holes. The proposed sensor structure is simple and presents very competitive sensing parameters, which demonstrates that this device is a promising alternative and could be used in a wide range of application areas.

## 1. Introduction

Surface plasmon polaritons (SPPs) are charge-density oscillations coupled to an electromagnetic field at a metal–dielectric interface, which can be modeled as propagation modes confined to the interface [1]. Following this thought, when a thin metal layer is sandwiched between two dielectric layers that have similar refractive indices, a dielectric–metal–dielectric waveguide (DMDW) is formed in which the SPP bound modes that are excited on the upper and lower surfaces of this waveguide can be coupled, creating symmetric and anti-symmetric modes, which are known as long-range SPPs (LRSPPs) and short-range SPPs (SRSPPs), respectively. LRSPPs have a longer propagation length and lower loss than SRSPPs [2,3,4]. The LRSPP modes have been observed experimentally [5,6], and due to their low loss properties they have a high potential for the development of applications based on planar waveguides, such as couplers, polarizers, perfect absorbers, sensors, nonlinear optics and solar energy [7,8,9,10,11,12], or LRSPP refractive index (RI) sensors based on prism-coupling, which use angular interrogation schemes [13] that use bulky optical components and require fine tuning, which make remote sensing and miniaturization difficult. Optical fibers with appropriate metal coating have also been demonstrated to be an SPP-sensing device. In most of the above cases, theoretical analysis is performed using analytical or semi-analytical mathematical models that exploit the characteristics of the simplified model of the planar waveguide, for instance, transfer matrix [14] or perturbation theory [15]. On the other hand, the emergence of microstructured optical fibers (MOFs) was one of the most important breakthroughs in the optical fiber sensor technology, since the optical characteristics of the propagating light into this kind of optical fibers and the evanescent field depend on the fiber microstructure and its geometrical parameters. Thus, the response of the devices based on MOFs can be controlled and improved trougth the variation of the geometrical structure, which allows more flexibility in the designs [16]. In recent years, many MOF-SPP sensors with different structures have been proposed to increase the sensitivity of these type of sensors, some of the alternatives are based on the selective filling of the fiber microstrusture with an analyte or with a metal nanowire [17,18]. In other alternatives, metallic layers are deposited on the inner walls of the microstructure holes then filled with analytes [19,20,21,22]. For an updated review of MOF-based SPP sensors see, e.g., [13]. Considering the limitations of the internal sensing mechanism (the metal coating and analyte are placed inside the fiber), SPP sensors based on D-shaped or exposed core structures provide a possible solution to internal sensing as the SPP is in direct contact with the external medium [23,24].

On the other hand, some designs of fiber-based SPP sensors use a high RI dielectric layer (TiO${}_{2}$) on top of the metal layer to facilitate spectral tuning towards larger wavelengths [25]. Even though these SPR sensors can achieve high sensitivities and low resolution values, the RI detection range is limited, and the values of loss at the SPP resonance are low.

Recent advances have shown other options to improve sensitivity [26,27]. Jiang et al., proposed a LRSPP sensor based on a dielectric/silver-coated hollow fiber that can detect high-RI liquid filling the hollow core of the fiber with an aqueous sample [21]. However, full width at half maximum (FWHM) of the SPP resonance of this sensor is large, which leads to low detection accuracy and resolution.

In this work, we propose and numerically analyze a simple structure to achieve LRSPP modes using an H-shaped optical fiber on which it is possible to deposit the DMDW structure using physical or chemical methods [26,28]. In this new configuration, the LRSPP modes interact directly with the analyte. Besides, the resonance wavelengths of LRSPP modes are shorter than SRSPP modes. Based on this approach, we present and discuss the performance of a novel design of a high sensitivity, multi-analyte RI sensor by LRSPP mode excitation in an H-shaped MOF with two symmetrical DMDWs deposited on the inner walls of its two side holes. Numerical simulations were performed using the finite element method (FEM) by taking into account the variations in the refractive index of the dielectric films, the thickness of the thin metal film, and the analyte RI changes. Our results indicate that the excitation of LRSPP modes in both open holes, but with different resonance wavelengths, have the potential to be operated either in self-reference mode or multi-analyte sensing applications. In addition, we found that varying the DMDW parameters is possible to obtain higher, narrower plasmonic peaks with similar intensities. Finally, we demonstrate that the deposition of the DMDW structure leads to operate at longer wavelengths, which is one of the main constraints of conventional plasmonic devices [29]. Thus, the proposed configuration based on DMDW improves significantly the key parameters of the device, such as the resolution (RES), sensitivity (S${}_{n}$), full width at half maximum (FWHM) and figure of merit (FOM).

## 2. Sensor Structure and Theory

**Figure 1:**
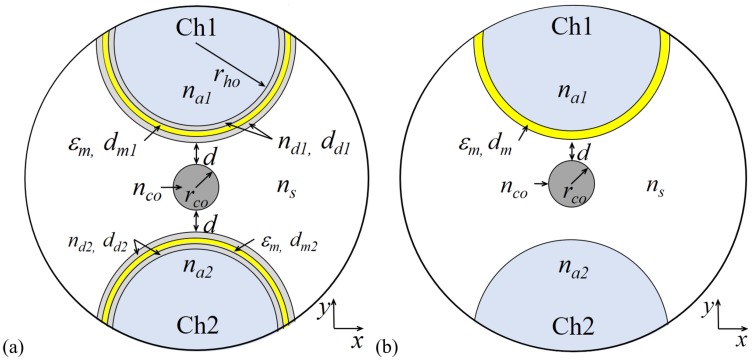
Structural parameters of the microstructured optical fiber (MOF): (**a**) with dielectric–metal–dielectric waveguide (DMDW) and (**b**) without DMDW.

**Figure 2:**
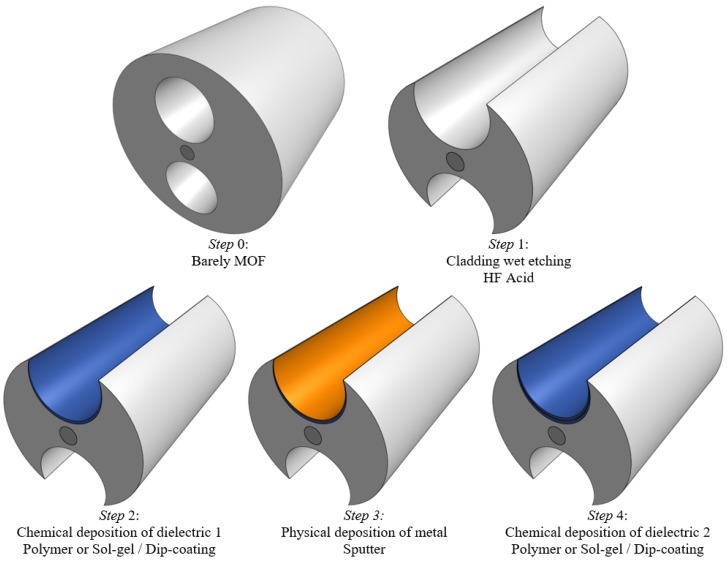
Schematic of the sensor fabrication process.

We propose a RI sensing device based on the structure shown in Figure 1a. It consists of an H-shaped silica MOF formed by a solid cladding of silica with refractive index $n_{s}$, a solid core of refractive index $n_{co}$ and radius $r_{co}$ = 2.5 μm, surrounded by two side holes of radius $r_{ho}$ = 15 μm, each one of them filled with a liquid analyte of refractive index $n_{aj},j=1,2$. Moreover, a symmetrical DMDW, formed by a thin gold layer of thickness $d_{m}$ and two dielectric layers of thickness $d_{d1}$ and $d_{d2}$, is deposited on the inner wall of the two open side holes at a distance *d*. Figure 2 shows the schematic of the sensor fabrication process. The RI of dielectric layers must be slightly higher than the fiber to control the coupling strength between the core mode and the plasmon wave, thus regulating the propagation loss of the sensor. The structure without DMDW, depicted in Figure 1b, is used as a reference and retains the optical properties of the fiber core, cladding, and thin metal layer.

**Figure 3:**
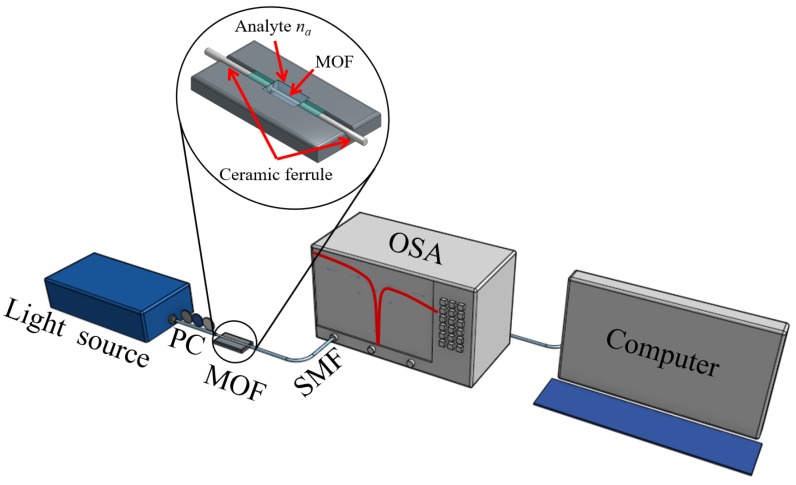
Proposed sensor setup for practical sensing. PC: Polarization controller; SMF: Single mode fiber; OSA: Optical spectrum analyzer.

Since the sensing region is exposed to the external environment in our design, the sensors can achieve rapid response and real-time sensing. A generalized sensing setup using the proposed sensor is shown in Figure 3. The single mode fiber (SMF) is used to launch the optical power from the broadband light source. A splicing technique can be used to connect the SMF with the proposed MOF sensor. The output spectra can be recorded over a large range of wavelengths using an optical spectrum analyser (OSA). The inset shows the design of a simple double v-groove holder with a small chamber reservoir in the center, which could be fabricated in glass, Teflon or other inert material to avoid chemical reactivity. The use of ceramic ferrules on the output ends of MOF facilitates the manipulation and interrogation of the device.

The modeling and numerical analysis were carried out using finite-element-method-based software COMSOL 5.1 The electromagnetic waves, frequency domain solver, with extremely fine element size, was used to obtain improved element quality. Quadratic triangular elements were chosen for improving the convergence and reducing the error. The maximum size element for the fiber core and dielectric layers was established as λ/5 (λ is the operating wavelength) and $d_{d}$/5, respectively, while in the metallic layer this parameter was established as $d_{m}/7$. A Cartesian perfectly matched layer (PML) with a width of 1.5 μm, a maximum mesh size of λ/5, and an outer scattering boundary condition, were used to avoid unwanted numerical reflections from the outer edge to the region under study. These provide the simulation accuracy of the analysis carried out for the proposed sensor.

The refractive indexes of the silica cladding $n_{s}$ and the doped silica core $n_{co}$ were modeled using the Sellmeier’s equation [30], while the Lorentz-Drude model [31] was used to model the electrical permitivity of metal $\varepsilon_{m}$. In this work the refractive indexes of dielectrics of DWDM $n_{d1}$ and $n_{d2}$, and the analite $n_{a}$ were considered variables.

In the DMDW structure, the symmetric plasmonic mode is localized near the dielectric with the lowest permittivity while the anti-symmetric mode is near dielectric with the highest permittivity [32]. On the other hand, the H-shaped fiber supports only one linearly polarized mode [22]. The propagation constants of the DMDW modes can be approximated using the following equation [33]

(1) $$\beta =\frac{2\pi }{\lambda }\sqrt{\frac{\varepsilon_{m}\varepsilon_{d}}{\varepsilon_{m}+\varepsilon_{d}}},$$

where $\varepsilon_{d}=n_{d}^{2}$ is the relative permittivity of the dielectric films of DWDM. Since these films are very thin and in contact with different media, $\varepsilon_{d}$ values can be considered averaged or weighted values [34], or effective values that are described in the effective-medium theory [35,36]; furthermore, the analyte RI is always lower than the cladding RI and hence, the symmetric plasmonic mode will be excited at the interface close to the analyte.

In accordance with the previous description, the operation principle of the sensor is based on the resonance of plasmonic modes excited by the fundamental core-guided mode. Clearly, the proposed structure is naturally suitable for a wavelength interrogation scheme [13,16,21,28,37,38]. Changes in the analyte RI are detected by measuring the shift of a resonance peak, $\lambda_{RES}$, in the loss spectrum of the core-guided mode, which can be obtained from the following equation,

(2) $$Loss\left(\lambda \right)=8.686\times \frac{2\pi }{\lambda }\times ℑ\left\{n_{eff}\right\}$$

where $ℑ\left\{n_{eff}\right\}$ is the imaginary part of the effective refractive index of the core-guided mode and λ is the wavelength in nm.

**Figure 4:**
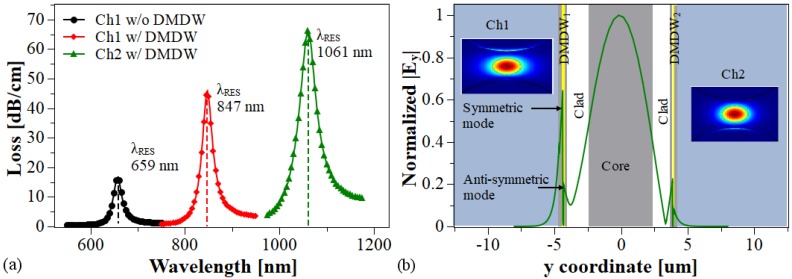
(**a**) Calculated loss spectra for the fiber core mode with $n_{a}$ = 1.36, *d* = 1 μm, $d_{m}$= 60 nm, $d_{d1}$ = 0.1 μm (Ch1), $d_{d1}$ = 0.2 μm (Ch2). (**b**) Normalized electric field.

Since the losses of the *x*-polarized mode are relatively low, only the *y*-polarized mode was considered in this study. For example, Figure 4a shows the resonance peaks under different conditions on the surface of the inner walls of the MOF. From this figure it is evident that the deposition of the DMDW improves the energy transfer from the core-guided mode to the symmetric plasmonic mode; consequently, we obtain higher resonant peaks with smaller spectral width. Also, the MOF with DMDW operates at larger wavelengths compared to the MOF without DMDW, which makes this sensor more promising as it will find significant applications in the field of biomedical at near IR region [29]. Figure 4b illustrates the normalized electric field distribution close to resonance condition. The insets in this figure confirm the largest energy transfers from the core-guided mode to the plasmonic mode with the DMDWs.

## 3. Sensor Performance and Discussion

**Figure 5:**
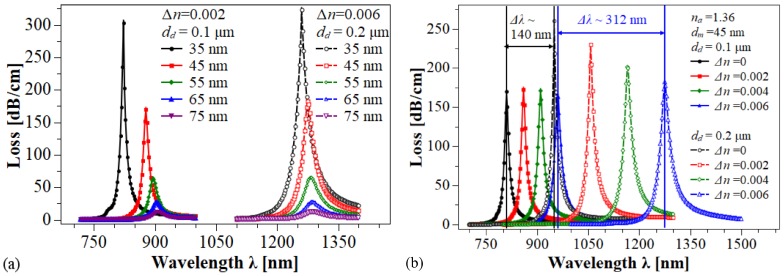
Loss spectra of the fiber core mode having a DMDW in each hole. (**a**) Two different values of $n_{d}$ and $d_{d}$, while varying the thickness $d_{m}$ of the gold layer. (**b**) Two different values of $d_{d}$, with $d_{m}$ = 45 nm, while increasing the refractive index (RI) of the DMDW dielectrics. *d* = 1 μm and $n_{a}$ = 1.36 were constant for all cases. Solid lines: Ch1. Dashed lines: Ch2.

Sensor performance was evaluated in terms of the resolution, RES = $\delta \lambda_{DR}$/S${}_{n}$, sensitivity, S${}_{n}$ = $\delta \lambda_{RES}/\delta_{na}$, full width at half maximum (FWHM) of the resonant peaks, $\Delta \lambda_{FWHM}$, and figure of merit, FOM = S${}_{n}/\Delta \lambda_{FWHM}$. $\delta_{na}$ describes the changes in the analyte refractive index, $\Delta \lambda_{RES}$ represents the shift in resonance wavelength, and $\Delta \lambda_{DR}$ is the minimal spectral resolution of spectrometer used to acquire the transmission loss spectrum. Figure 5a depicts the behavior of the loss spectra for two different configurations of the DMDW having different values of Δn and $d_{d1}$, while the thickness of gold layer $d_{m}$ was varied from 35 nm to 75 nm; $n_{a}$ = 1.36 was assumed to be constant. As can be seen, in both cases the increase in $d_{m}$ decreases the strength of the resonance peak and shifts the resonance wavelength towards longer wavelengths. On the other hand, Figure 5b shows the loss spectra for two different values of $d_{d}$. Here, $n_{d}$ was changed from $n_{d}=n_{co}$ to $n_{d}=n_{co}$ + 0.006 RIU in steps of 0.002 RIU, while $n_{a}$ and $d_{m}$ were kept constant at 1.36 RIU and 45 nm, respectively. In both cases, we observe a redshift in the resonance wavelength with the increase in Δn, however, this shift is greater in the structure with the highest value of $d_{d}$. It is important to note that the variations in Δn did not generate changes in the shape of the loss spectrum, unlike what is observed when the thickness of the gold layer was varied.

**Figure 6:**
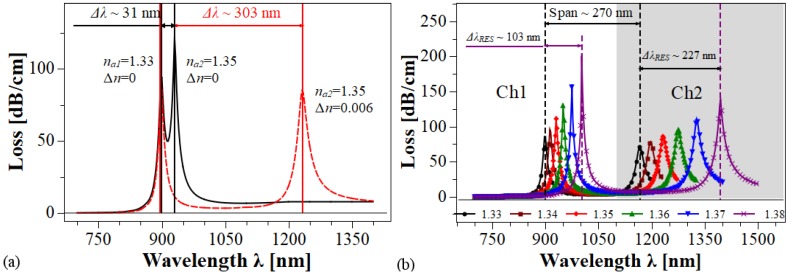
(**a**) Self-referencing and/or multi-analyte operation with $d_{m}$ = 45 nm, d=1μm. Black line: $d_{d1}=d_{d2}$ = 0.1 μm. Red line: $d_{d1}$ = 0.1 μm, $d_{d2}$ = 0.2 μm. (**b**) Loss spectra with different values of $n_{a}$ in Ch1 and Ch2. $d_{m}$ = 45 nm, *d* = 1 μm, $d_{d1}$ = 0.1 μm, $\Delta n_{1}$ = 0; $d_{d2}$ = 0.2 μm, $\Delta n_{2}$ = 0.006.

In addition, the potential of the structure in self-referenced or multi-analyte operation was investigated. Two different cases were considered. In the first one, the DMDWs deposited inside each side hole of the MOF maintain their geometrical and optical parameters. As is shown in Figure 6a, in this case the shift in the resonant wavelength is only a few dozen nanometers and, therefore, the two loss spectra overlap, making difficult to resolve the resonance-wavelength shifts. In the other case, the DMDWs deposited have similar geometries, i.e., $d_{d1}$ = $d_{d2}$ and $d_{m1}$ = $d_{m2}$, but the RI of the DMDW dielectric $n_{d1}$ and $n_{d2}$ are different. In this case, depicted in the Figure 6b, the peaks of loss spectra appear sufficiently separated, about ten times the shift of the previous condition, and therefore could easily be resolved. Also, the strength of resonance peaks was similar, which facilitates their detection. This characteristic in the sensor spectral response allows multi-analyte detection since the shifts of the loss peak in Ch1, controlled by variations of $n_{a1}$, are independent of the shifts of the loss peak in Ch2, controlled by variations of $n_{a2}$. In both cases, the fiber holes were filled with analytes $n_{a1}$ = 1.33 and $n_{a2}$ = 1.35, respectively.

As it is shown in Figure 6b, by combining the effects caused by the variations in the geometrical and optical parameters of the DMDWs, mainly in thicknesses $d_{d1}$ and $d_{d2}$, and increases in the values of refractive index $\Delta n_{1}$ and $\Delta n_{2}$ of the dielectric layers, it is possible to obtain configurations in which two LRSPPs are excited at different ranges of wavelength and, consequently, for each value of $n_{a}$ two well defined loss peaks appear in the loss spectrum. Note that the separation between the two resonance wavelengths for the lower value of $n_{a}$ is larger than $\Delta \lambda_{RES}$ that occur when $n_{a}$ changes from a lower to a higher value. Moreover, this behavior could be used to develop an auto-referenced sensor, in which the analyte RI inside one of the side holes, for example, $n_{a1}$ is maintained at a value constant, while the analyte RI inside the other side hole $n_{a2}$ is varied.

**Figure 7:**
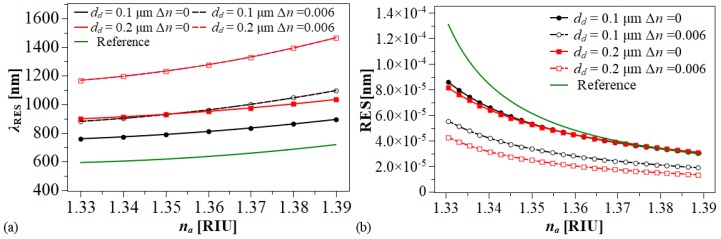
Operation of the RI sensor based on long-range surface plasmon polaritons (LRSPPs). (**a**) $\lambda_{RES}$, (**b**) S_n_ with $d_{m}$ = 30, nm, *d* = 1μm. Black lines: Ch1. Red lines: Ch2. Green line: Ch1 without DMDW (Reference).

Based on the above, the sensor performance for each hole was analyzed separately. Figure 7a shows that, for all the cases studied, the resonant wavelength shifts exhibit a nonlinear feature due to the higher value of $n_{a}$ enable a strong mode coupling between the fundamental core-guided mode and the LRSPP mode. Figure 7b illustrates the sensor sensitivity S${}_{n}$ as a function of the analyte RI. The sensitivity ranges are 1170 nm/RIU to 3300 nm/RIU and 2370 nm/RIU to 7540 nm/RIU for the DMDW with $n_{d}=n_{co}$ and for the DMDW with $n_{d}=n_{co}$ + 0.006, respectively. In addition, from Figure 7b it is evident that the sensitivity of the proposed structure depends on the value of refractive index of dielectric layer $n_{d}$ in the DMDW.

**Figure 8:**
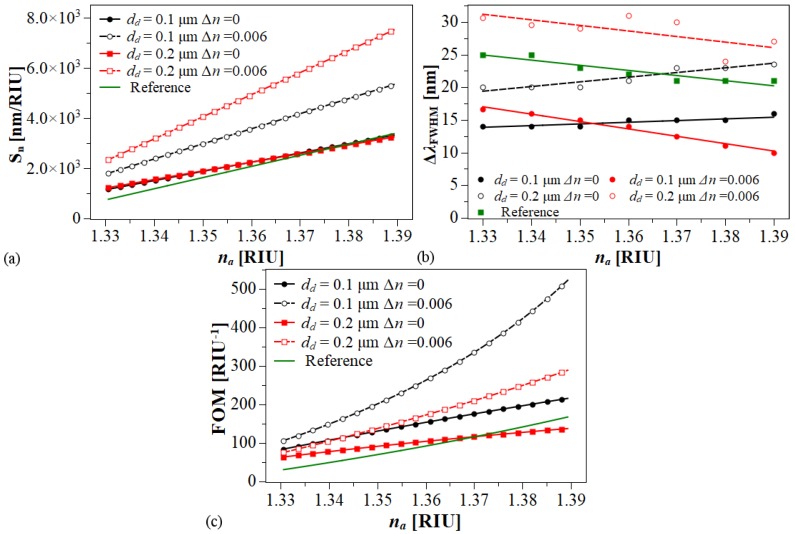
Performance of the RI sensor based on LRSPP. (**a**) S${}_{n}$, (**b**) FWHM and (**c**) FOM with $d_{m}$ = 30, nm, *d* = 1 μm. Black lines: Ch1. Red lines: Ch2. Green line: Ch1 without DMDW (Reference).

On the assumption that a 0.1 nm resonance wavelength peak can be detected, the sensor resolution is RES = 0.1/S${}_{n}$. Therefore, the minimum resolutions that can be achieved for the above conditions are 3.0 × 10${}^{-5}$ RIU and 1.3×10${}^{-5}$ RIU, respectively, as shown in Figure 8a.

Although the attained maximum sensitivity is not as high as the refractive index sensors based on sophisticated MOFs [20,21,23,24], which as mentioned above demand great technical efforts for their fabrication, we aim to provide new ideas for the development of optical fiber SPP sensors. Here, alternatively, we present a sensor characterized by its simplified microstructure based on the long-range surface plasmon excitation, which may be meaningful for the SPP sensor research. Figure 8b illustrates the simulation results obtained for the $\Delta \lambda_{FWHM}$. In each case, they were estimated by fitting the spectral loss to a Gaussian function. The behavior of this parameter has an apparent dependence on the thickness of DMDW dielectric layer $d_{d}$. The lowest calculated values were 15.3 nm and 10.3 nm. Despite the fact that thicker films increase the $\Delta \lambda_{FWHM}$, it is also clear that by increasing the refractive index of dielectric films it is possible to significantly improve the S${}_{n}$ values and, consequently, as shown in Figure 8c, the FOM is significantly enhanced. According to the above results, it is important to emphasize that variations on the values of $n_{d}$ and $d_{d}$ have important effects on sensor performance and, therefore, by controlling them is possible to tune the sensors response.

As can be seen in Table 1, the results obtained in this work show that sensors based on LRSPP are competitive and outstanding among numerous MOF-based SPP sensor alternatives because it offers a large dynamic detection range, competitive resolution, and improvements in sensitivity and FOM without intricate designs and whose manufacture is limited to achievable DMDW structure.

## 4. Conclusions

In summary, we have proposed a novel high sensitivity RI sensor based on LRSPP resonance in H-shaped MOF with symmetrical DMDW. The presence of the DMDW enables the excitation of symmetric and antisymmetric plasmonic modes and facilitates the coupling between the fiber core mode and the symmetric plasmonic mode, improving the sensor parameters. It has been shown that an analyte RI range from 1.33 to 1.39 can be achieved as well as sensitivities of up to 7540 nm/RIU, with resolution values as low as 1.3 × 10${}^{-5}$ RIU and a FOM value of 522 RIU${}^{-1}$ at $n_{a}$ = 1.39. Also, the proposed structure could be used to implement two functionalities: multi-analyte sensing and self-referencing. The operation in the multi-analyte mode enables the detection of two analytes in one aqueous sample. It can also be used in the identification of two different liquid samples with high sensitivity. The results of this work can have a significant impact on the design of MOF-based SPP sensors aimed at striking a good balance between sensor performance and ease of manufacture.

## Figures and Tables

**Table 1:** Comparison of recently reported fiber-optic refractice index sensors based on SPP and LRSPP.

Description	RI Range	$S_{n}$ (nm/RIU)	*FWHM* (nm)	*RES* (RIU)	*FOM* (RIU${}^{-1}$)	Ref.	Year
SPR sensor based on H-shaped fiber with high RI dielectric layer (TiO${}_{2}$) on top of the metal layer.	1.32 to 1.33	5100	NA *	NA *	NA *	[25]	2011
LRSPR sensor based on dielectric/silver coated hollow fiber.	1.518 to 1.576	6600	100	1.51 × 10${}^{-5}$	78	[21]	2015
Fiber optic SPR sensor based on MMF-FBG-MMF structure.	1.333 to 1.380	2557	170	0.2 × 10${}^{-6}$	15	[39]	2016
SPR-based PCF sensor with NLC core.	1.33 to 1.34	3900	NA *	2.56 × 10${}^{-5}$	NA *	[40]	2017
LRSPR sensor using a side polished fiber with the buffer layer of magnesium fluoride.	1.33 to 1.38	3628	34	2.75 × 10${}^{-5}$	154	[41]	2017
RI sensor based on a D-shaped PCF with a nanoscale gold belt.	1.2 to 1.4	3751	NA *	1 × 10${}^{-5}$	NA *	[42]	2018
SPR-based D-shaped single mode fiber sensor with a gold grating over the polished fiber surface.	1.33 to 1.34	7590	NA *	1.31 × 10${}^{-5}$	NA *	[43]	2019
LRSPR sensor based on GK570/Silver coated hollow fiber (HF) with an asymmetric layer structure.	1.4772 to 1.5116	12,500	83	0.8 × 10${}^{-5}$	150	[44]	2019
A D-shaped Fiber LRSPR Sensor with High Q-factor.	1.332 to 1.382	3627.51	81	2.76 × 10${}^{-7}$	53	[45]	2019
A fiber-based symmetrical LRSPR biosensor with high Q-Factor.	1.33 to 1.38	3499	76	2.86 × 10${}^{-7}$	46	[46]	2019
LRSPR in H-shaped MOF with symmetrical dielectric–metal–dielectric waveguide.	1.33 to 1.39	7540	27	1.3 × 10${}^{-5}$	280	This work	

* The authors do not provide this information.
